# In situ dissecting the evolution of gene duplication with different histone modification patterns based on high-throughput data analysis in *Arabidopsis thaliana*

**DOI:** 10.7717/peerj.10426

**Published:** 2021-01-05

**Authors:** Jingjing Wang, Yuriy L. Orlov, Xue Li, Yincong Zhou, Yongjing Liu, Chunhui Yuan, Ming Chen

**Affiliations:** 1 Center for Stem Cell and Regenerative Medicine, The First Affiliated Hospital, Zhejiang University School of Medicine, Zhejiang University, Hangzhou, P. R. China; 2 Department of Bioinformatics, The State Key Laboratory of Plant Physiology and Biochemistry, Institute of Plant Science, College of Life Sciences, Zhejiang University, Hangzhou, P. R. China; 3 James D. Watson Institute of Genome Sciences, Zhejiang University, Hangzhou, P. R. China; 4 Zhejiang Provincial Key Lab for Tissue Engineering and Regenerative Medicine, Dr. Li Dak Sum & Yip Yio Chin Center for Stem Cell and Regenerative Medicine, Zhejiang University, Hangzhou, P. R. China; 5 The Digital Health Institute, I.M Sechenov First Moscow State Medical University (Sechenov University), Moscow, Russia; 6 Novosibirsk State University, Novosibirsk, Russia; 7 Agrarian and Technological Institute, Peoples’ Friendship University of Russia (RUDN), Moscow, Russia; 8 Institute of Hematology, Zhejiang University, Hangzhou, P. R. China; 9 Key Laboratory of Structural Biology of Zhejiang Province, School of Life Sciences, Westlake University, Hangzhou, Zhejiang Province, P. R. China

**Keywords:** Histone modifications, Plant genome, Bioinformatics, Gene duplication, Epigenetic pattern evolution, ChIP-chip, *A. thaliana*, RNA-seq

## Abstract

**Background:**

Genetic regulation is known to contribute to the divergent expression of duplicate genes; however, little is known about how epigenetic modifications regulate the expression of duplicate genes in plants.

**Methods:**

The histone modification (HM) profile patterns of different modes of gene duplication, including the whole genome duplication, proximal duplication, tandem duplication and transposed duplication were characterized based on ChIP-chip or ChIP-seq datasets. In this study, 10 distinct HM marks including H2Bub, H3K4me1, H3K4me2, H3K4me3, H3K9ac, H3K9me2, H3K27me1, H3K27me3, H3K36me3 and H3K14ac were analyzed. Moreover, the features of gene duplication with different HM patterns were characterized based on 88 RNA-seq datasets of *Arabidopsis thaliana*.

**Results:**

This study showed that duplicate genes in *Arabidopsis* have a more similar HM pattern than single-copy genes in both their promoters and protein-coding regions. The evolution of HM marks is found to be coupled with coding sequence divergence and expression divergence after gene duplication. We found that functionally selective constraints may impose on epigenetic evolution after gene duplication. Furthermore, duplicate genes with distinct functions have more divergence in histone modification compared with the ones with the same function, while higher expression divergence is found with mutations of chromatin modifiers. This study shows the role of epigenetic marks in regulating gene expression and functional divergence after gene duplication in plants based on sequencing data.

## Introduction

Gene duplication plays a critical role in the generation of genomic repertoires for subsequent functional innovation (Flagel & Wendel, 2009). Duplicate genes can be generated through different molecular processed modes, such as whole genome duplication (WGD) and segmental duplication (SD) (Freeling, 2009; Kustatscher et al., 2019; Wang, Tan & Paterson, 2013). WGD is induced by abnormal cell division, which is a common phenomenon in the plant kingdom (Otto & Whitton, 2000; Zhao et al., 2015). Single-gene duplications are classified as local duplications (including tandem duplication and proximal duplication), which could generate two nearby duplicate genes, and transposed duplications, which could create a gene duplication with a copy of the transposable element at a new chromosomal position via either DNA or RNA-based mechanisms (Cusack & Wolfe, 2007; Wang, Tan & Paterson, 2013; Wang, Wang & Paterson, 2012).

Following gene duplication, retained paralogs initially have identical sequences and functions but tend to undergo divergence in DNA sequence and expression patterns later (Liao et al., 2014). Growing evidence suggests that duplicated genes are regulated by both genetic and epigenetic regulation (Chen, 2007). A variety of molecular mechanisms of epigenetic regulation has been described, with the most important being histone modification (HM), DNA methylation and histone variants. Histone modification is a covalent post-translational modification to histone proteins, which impact gene expression by altering chromatin conformation or recruiting chromatin modifiers and transcriptional regulators. In most species, histone proteins contain four types: H2A, H2B, H3 and H4, which are subject to post-translational modifications including acetylation, methylation, phosphorylation and ubiquitylation.

Histone modifications may occur in a locus-specific manner. For example, histone H3 is primarily acetylated (ac) at Lysines (K) 9, 14, 18, 23 and 56, methylated (me) at Lysines (K) 4, 9, 27, 36 and 79, and phosphorylated at Ser10, Ser28, Thr3 and Thr11. H3K4me1 is an epigenetic modification to protein Histone H3 and it is a mark that indicates the mono-methylation at the fourth lysine residue of H3. Histone modification is an important type of epigenetic marks which could affect the gene transcriptional activation. Some marks are located in the vicinity of activated genes, such as H3K4me1/2/3, H3K9ac, H3K14ac and H3K36me3; these marks are called active marks. Some marks are located next to repressed genes, such as H3K27me1/3; these marks are called repressive marks. Our previous study has discussed the distribution dynamics and roles of epigenetic marks in the modulation of gene transcription (Wang et al., 2015b). Several lines of evidence suggest that epigenetic changes play an important part in the initial expression divergence between duplicated gene pairs. Zou et al. (2012) have investigated the HM pattern evolution after gene duplication in yeast and showed that genetic and epigenetic elements, which contribute together to the expression divergence between duplicated gene pairs, have co-evolved since gene duplication. The expression levels of three TaEXPA1 homologs were correlated to the change with H3K9me3, H3K4me3 and H3K9ac in hexaploid wheat (Hu et al., 2013). It has been reported that DNA methylation divergence increases with evolutionary age between the duplicate gene pair in human (Keller & Yi, 2014). Also, DNA methylation is proposed as a potentially important regulatory mechanism for gene expression divergence in plants (Sui et al., 2014; Wang, Marowsky & Fan, 2014). Specifically, exonic methylation divergence correlates more closely with expression divergence than intronic methylation divergence (Wang, Marowsky & Fan, 2014). In our previous study, we found that the DNA methylation of the open reading frame region (ORF methylation) illustrates distinct patterns in different modes of duplication (Wang et al., 2013). Furthermore, researchers suggested that duplicated genes with the most divergent ORF methylation and expression patterns tended to have distinct biological functions in cassava (Wang et al., 2015a). It was also reported that the important feature of the changes in ORF methylation of duplicated genes in *A. thaliana* is to undergo evolutionary transormation (Wang, Marowsky & Fan, 2014; Wang & Adams, 2015).

Berke & Snel (2014) have found that H3K27me3 is retained and constrains expression divergence after duplication in *A. thaliana* genomes, which is linked to lower expression divergence, yet higher coding sequence divergence (Berke, Sanchez-Perez & Snel, 2012). However, it remains to be explored whether and how the comprehensive HM profiling, not just a single chromatin mark, influences the expression of duplicate genes in *A. thaliana*. In this work, we aim to investigate the potential interplay between the HM profiling and gene duplications in *A. thaliana*, which can help us to understand the mechanisms underlying gene duplication, evolution, and the roles of HM marks in shaping current genomes (Wang et al., 2013).

## Materials and Methods

### Identification of duplicate genes with different modes in *A. thaliana*

The MCScanX-transposed software is commonly used to identify gene duplications that occurred within different areas based on the MCScanX algorithm which can detect multiple gene collinearity within and between related genomes (Wang et al., 2013). We applied MCScanX-transposed software with default parameters to identify duplicated genes with different modes, including WGD, proximal, tandem and transposed duplications in the *A. thaliana* genome. The unidentified genes were considered as singleton genes.

### Histone profiling

Public ChIP-chip data (Dataset S2) were originally mapped to the TAIR10 version of the *A. thaliana* reference genome. A total of 10 distinct HM marks including H2Bub, H3K4me1, H3K4me2, H3K4me3, H3K9ac, H3K9me2, H3K27me1, H3K27me 3, H3K36me3 and H3K14ac were analyzed (Additional file 4). For each of the ten HM marks in the open reading frame regions (ORF) and upstream regulatory (promoter) regions (500 bp upstream of the transcription start site), we calculated the log2-transformed average enrichment ratio with nucleosome normalizing. The ratios were then transformed to z scores using the formula *Z*_X_ = (χ − μ)/δ, where χ is the ratio value for a gene, μ is the mean ratio of all genes, and δ is the standard deviation of this ratio for all genes. The HM divergence of a duplicate gene pair was measured by *D*_HM_ = 1− *r*, where *r* is the Pearson correlation coefficient of the HM profiles for the duplicated gene pair.

### Randomized controlled analysis

Randomization tests examine data in a nonparametric way, without the need to make assumptions about object populations. The most important advantage of randomization tests is that they allow users to specify any test statistic of interest and remain statistically valid. To test whether the duplication group of interest is significantly diﬀerent from others, the randomization test is applied.

For each test, 10,000 independent randomizations are performed for the controlled analysis. Note that, (i) to obtain the randomized control of HM divergence between duplicate pairs, we randomly generated the same number of singleton pairs. (ii) To test the chromosome bias on the HM divergence between duplicate pairs, randomized singleton pairs were chosen where both copies were located on the different chromosomes. Then, the Wilcoxon rank-sum test was used to evaluate the significance of distribution between the randomized control and observed data sets.

### Statistical method

To compare the chromosome bias of HM divergence in ORF and promoter regions of different modes of gene duplication, we calculated the *D*_HM_ and compared the mean values of *D*_HM_ in each mode. Wilcoxon rank-sum test was applied to calculate the significance. The same method was applied to assess whether the patterns of expression divergence of duplicated genes were consistent across different perturbation conditions or not.

### *K*_S_ and *K*_A_ calculation

Coding sequence divergence was measured by the rate of synonymous substitutions (*K*_S_) and nonsynonymous substitutions (*K*_A_). *K*_S_ and *K*_A_ between duplicated gene pairs were estimated using MCScanX-transposed software with default parameters.

### Gene expression analysis

A total of 88 RNA-seq samples (Dataset S1) from 16 publications were divided into three sample conditions based on different perturbation conditions: (i) the normal development group, named as “Normal” containing 18 wild-type samples; (ii) the group of samples with environmental stresses, named as “Stress” containing 20 samples; (iii) the group of samples with mutations on chromatin modifier (CM), named as “CM_mu” containing 50 samples. These RNA-seq datasets were re-analyzed according to the protocol proposed by Trapnell et al. (2012). This protocol used TopHat2, which is a fast splice junction mapper for RNA-Seq reads (Kim et al., 2013) and Cufflinks2, which could assemble transcripts, estimate their abundances, and test for differential expression based on aligned RNA-Seq reads (Trapnell et al., 2010). Here, the raw datasets were downloaded from the SRA database according to the corresponding SRA ID listed in Additional file 1. Then the reads were aligned to TAIR10 using TopHat2. Finally, the gene expressed abundances were calculated using Cufflinks2.

### Functional analysis

To understand the biological function of duplicate genes, the Database for Annotation, Visualization and Integrated Discovery (DAVID) tool with version 6.7 (Huang, Sherman & Lempicki, 2009) was applied to identify the enriched Gene Ontology (GO) functional terms based on each mode of the gene duplication. Further, REVIGO, which could summarize the long lists of GO terms by removing redundant terms (Supek et al., 2011), was used to remove redundant GO terms and visualize the GO enrichment results. All genes and its annotated standard GO terms of arabidopsis are listed in Additional file 5.

## Results

### Identifying different modes of duplicate genes

**Figure 1:**
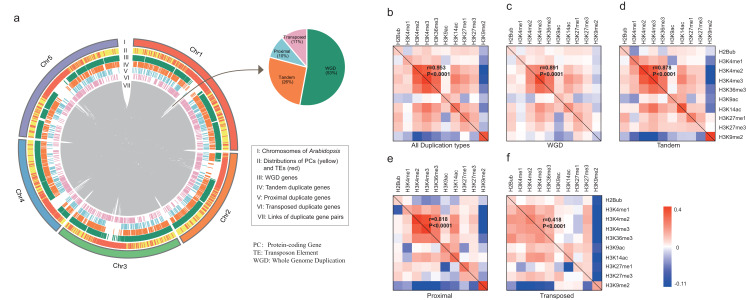
Characterization of duplicate genes in *A. thaliana*. (A) The Circus diagram represents the distribution of four types of gene duplication. From outermost to innermost, the first track represents *A. thaliana* chromosomes, the second track represents the distribution of PGs (yellow) and Transposable elements (TEs, red), the third, fourth, fifth and sixth tracks show the distributions of WGD, tandem, proximal and transposed duplicate genes, respectively, innermost link demonstrates the paralog pairs’ loci in chromosomes and inset pie chart represents the distribution of duplicate gene pairs in four types. Heatmap matrix of pairwise Pearson correlation of histone modification marks for different types of paralog pairs in ORF regions: all duplication types (B), WGD (C), Tandem (D), Proximal (E) and Transposed (F) For a given paralog pair (gene A and B), the upper right diagonal shows the correlation coefficients based on gene A; the lower-left diagonal shows the correlation coefficients based on gene B. The correlations of overall histone modification profiles between paralog pairs were calculated with Mantel test.

All *A. thaliana* genes were classified by four modes of gene duplication including WGD, proximal, tandem and transposed duplication, and singleton genes (“Materials and Methods”). According to the definition, 8,111 duplicate gene pairs were identified including 4,293 (53%) WGD gene pairs, 2,130 (26%) tandem duplicate gene pairs, 784 (10%) proximal duplicate gene pairs and 904 (11%) transposed duplicate gene pairs (Dataset S2). As shown in Fig. 1A, duplicate genes are scattered across the whole genome, whereas only transposed duplicate genes tend to be located in transposable elements (TEs)-enriched region.

We then calculated the enrichment levels of each HM mark for each gene within its ORF and promoter region (“Materials and Methods”, Figs. S1 and S2). Comparing with singletons, WGD genes were more likely to be targeted by active marks (including H3K4me1/2/3, H3K9ac, H3K14ac and H3K36me3) and H3K27me1/3, instead of H3K9me2 in both ORF and promoter regions (Wilcoxon rank-sum test: *p*-value < 0.05 for all cases). Additionally, transposed duplicate genes show a higher level of H3K9me2 than other modes of duplicate genes and singletons (Wilcoxon rank-sum test: *p*-value < 0.05 for all cases).

Then, we calculated the pairwise Pearson correlations of HM marks for paralog pairs. The result showed that the correlation coefficients between the duplicate gene pairs for each mark are positive, except for the coefficients between WGD pairs for H3K9me2 (Table 1). Moreover, these correlation coefficients in promoter regions are larger than in ORF regions. On the other hand, some histone modification marks showed co-occurrence between duplicate gene pairs, and the overall patterns of histone modification marks between duplicate pairs are consistent (Pearson correlation coefficient: 0.418 < *r* < 0.991 and *p*-value < 1.0E−4, Mantel test (Mantel, 1967), Figs. 1B–1F and Fig. S3).

**Table 1:** The Pearson correlation between paralog pairs.

Mark		All duplication		WGD		Tandem		Proximal		Transposed	
		ORF	Promoter	ORF	Promoter	ORF	Promoter	ORF	Promoter	ORF	Promoter
H2Bub	*r*	0.072	0.180	0.052	0.125	0.089	0.257	0.074	0.254	0.003	0.138
	*p*-value	7.15E−11	2.60E−60	6.79E−04	1.63E−16	3.79E−05	1.57E−33	0.039	5.76E−13	9.23E−01	3.11E−05
H3K4me1	*r*	0.137	0.545	0.081	0.570	0.236	0.609	0.149	0.512	0.128	0.303
	*p*-value	2.63E−35	0	9.72E−08	0	2.03E−28	5.49E−216	2.93E−05	1.47E−53	1.16E−04	1.19E−20
H3K4me2	*r*	0.339	0.582	0.215	0.489	0.434	0.643	0.482	0.624	0.244	0.393
	*p*-value	7.94E−218	0	7.05E−46	2.23E−257	8.74E−99	4.68E−249	7.52E−47	5.62E−86	1.00E−13	7.97E−35
H3K4me3	*r*	0.445	0.620	0.386	0.534	0.449	0.657	0.403	0.618	0.262	0.402
	*p*-value	0	0	7.51E−153	1.26E−321	2.38E−106	1.12E−263	6.41E−32	7.11E−84	1.10E−15	1.62E−36
H3K36me3	*r*	0.268	0.528	0.248	0.442	0.263	0.519	0.250	0.490	0.164	0.392
	*p*-value	3.24E−133		4.37E−61	8.77E−205	6.03E−35	3.08E−147	1.18E−12	1.38E−48	7.71E−07	1.29E−34
H3K14ac	*r*	0.135	0.359	0.113	0.340	0.173	0.460	0.216	0.387	0.081	0.221
	*p*-value	1.83E−34	1.60E−245	1.32E−13	1.47E−116	8.55E−16	5.23E−112	1.02E−09	1.73E−29	1.46E−02	1.70E−11
H3K9ac	*r*	0.270	0.463	0.204	0.405	0.349	0.553	0.285	0.433	0.147	0.294
	*p*-value	3.46E−135	0	2.14E−41	3.15E−169	5.48E−62	7.22E−171	3.76E−16	4.13E−37	8.68E−06	1.69E−19
H3K27me1	*r*	0.227	0.376	0.157	0.241	0.244	0.459	0.267	0.473	0.119	0.308
	*p*-value	6.21E−95	6.92E−271	4.75E−25	1.33E−57	2.55E−30	3.11E−111	2.86E−14	6.85E−45	3.46E−04	2.71E−21
H3K27me3	*r*	0.107	0.152	0.081	0.115	0.085	0.216	0.127	0.214	0.065	0.100
	*p*-value	3.86E−22	5.35E−43	9.49E−08	4.76E−14	7.82E−05	7.77E−24	3.71E−04	1.40E−09	5.20E−02	2.50E−03
H3K9me2	*r*	0.330	0.369	−0.019	−0.023	0.831	0.902	0.788	0.847	0.134	0.201
	*p*-value	2.40E−205	1.10E−259	2.07E−01	0.128	0	0	4.23E−167	2.90E−217	4.98E−05	1.10E−09

**Notes:**

*r* indicates the Pearson correlation coefficient.

*p*-value indicates the significance of Pearson correlation coefficient.

### Divergence of histone modification profiles among different modes of duplicate genes

We further examined the divergence of HM profile between paralog pairs and singletons. The divergence of HM profiles was calculated (“Materials and Methods”) in both ORF and promoter regions, which were denoted by scores: *D*_HM–O_ and *D*_HM–P_, respectively. To compare the HM divergence between paralog pairs and randomized singleton pairs, we randomly selected 8,111 pairs from singletons with 10,000 repeats. We found that both *D*_HM–O_ and *D*_HM–P_ are lower between duplicate pairs than that of randomized pairs (Wilcoxon rank-sum test: *p*-value < 2.2E−16 for both cases), which is consistent with the previous study in yeast (Zou et al., 2012).

**Figure 2:**
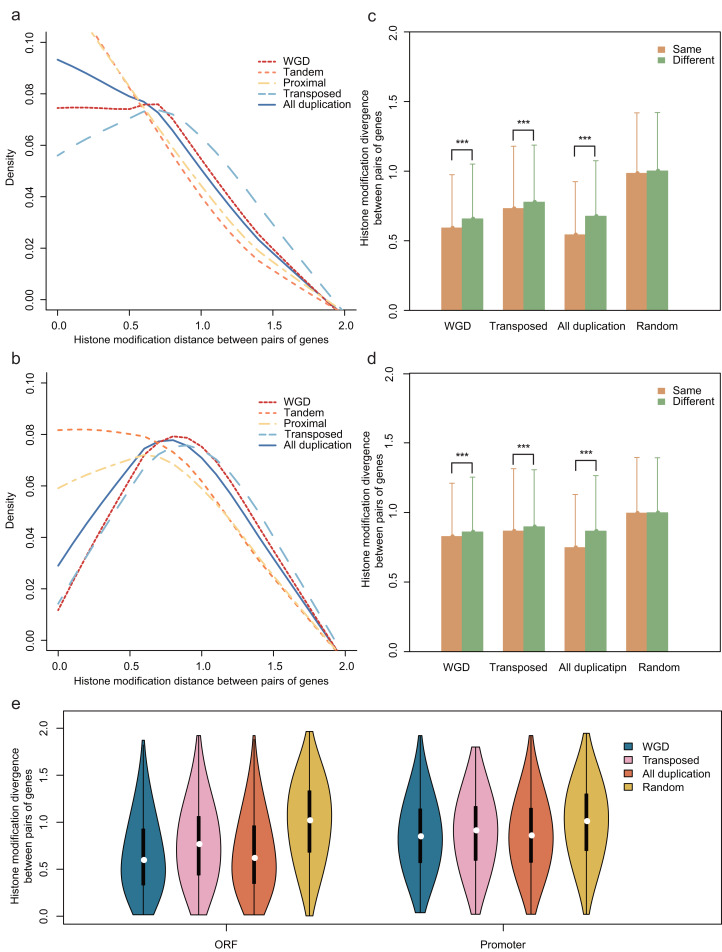
Comparison of the HM divergence between paralog pairs in *A. thaliana*. The distribution of HM divergence among WGD, tandem, proximal, transposed and total duplicate genes (duplicate) in ORF (A) and promoter regions (B). Comparison of chromosome bias on HM divergence among different types of gene duplication in ORF (C) and promoter regions (D). Here, chromosome bias represents that two genes of a duplicate gene pair tend to be located on the same chromosome. Significance difference between same and different chromosomes for HM distance are indicated with “***” for *P-value* < 0.001. (E) Comparison of HM divergence between singleton pairs and paralog pairs on different chromosomes in ORF and promoter regions.

To investigate the HM status of different gene duplication regions, we compared the effects of HM profile on different gene regions of each gene duplication mode. The results showed that the divergence of HM profile both in ORF and promoter regions are distinct among the different modes of gene duplication (Kruskal-Wallis Test, *p*-value < 2.2E−16; Figs. 2A and 2B). Notably, the transposed duplicates have a higher divergence score than other duplicate modes, meanwhile, tandem and proximal duplicates have lower divergence score.

The physical distance between single-gene duplications followed a trend: transposed duplicates > proximal duplicates > tandem duplicates. We assumed that position effects may have an influence on the divergence of HM profiles of genes with different duplication modes. To test this hypothesis, we selected all *A. thaliana* gene pairs on the same chromosomes and computed the correlation between HM divergences and physical distances of gene pairs both in ORF and promoter regions. Notably, the HM divergence of gene pairs on the same chromosome is not significantly correlated with physical distance (Pearson correlation coefficient: *r* = 0.066 for ORF regions and *r* = 0.089 for promoters). However, gene duplicate pairs on the same chromosome share a more similar HM profile than that on different chromosomes in ORF and promoter regions (Wilcoxon rank-sum test: *p*-value < 2.2E−16 for both cases, Figs. 2C and 2D).

Notably, duplicate pairs tend to be located on the same chromosome comparing with randomized singleton pairs (Chi-squared test: χ^2^ = 152.0918, df = 1, *p*-value < 2.2E−16), which is termed as “chromosome bias”. This falls in line with the research in yeast (Zou et al., 2012). We then wanted to know whether chromosome bias gives a possible explanation for less difference of HM divergence between the duplicate gene pairs than that between randomized singleton pairs. To rule out the possibility, the same number of duplicate gene pairs and random pairs were selected in which both copies located on different chromosomes, with a result that HM divergence was lower between paralog pairs than that between randomized singleton pairs (Fig. 2E, Student’s *t*-test: *p*-value < 0.001 for both ORF and promoter regions).

### Histone modification divergence of paralog pairs along with evolutionary time

**Figure 3:**
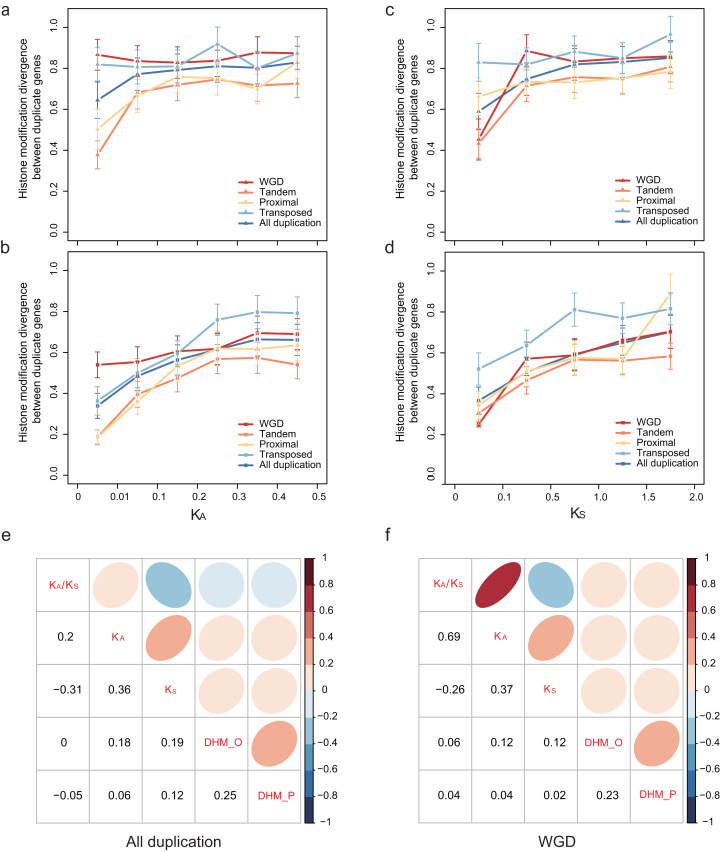
Relationship between patterns of HM marks and the evolutionary ages of duplicate genes. HM divergence between paralog pairs increases with duplicate genes nonsynonymous distance *K*_A_ in promoters (A) and ORF regions (B), and with duplicate genes synonymous distance *K*_S_ in promoters (C) and ORF regions (D). Correlation matrix between HM divergence and sequence divergence for all duplicate genes (E) and WGD genes (F). For other types of duplication, see Fig. S4. ‘D_HM_O_’ represents the HM divergence in ORF regions; ‘D_HM_P_’ represents the HM divergence in promoter regions.

To explore how HM profile evolves following gene duplication, the relationship of the HM profile of duplicate genes and the divergence of their coding sequence was characterized. In our study, coding sequence divergence was measured by synonymous substitutions (*K*_S_) rate and nonsynonymous substitutions (*K*_A_) rate. The duplicate pairs with *K*_A_ < 0.5 and *K*_S_ < 2.0 were selected for further analysis. We related the divergence of HM profile of duplicate genes to *K*_A_ (Figs. 3A and 3B) and *K*_S_ (Figs. 3C and 3D) using a scatterplot. We observed that *D*_HM–O_ and ${D_{\rm HM - P}}$ are weakly positively correlated with *K*_S_ and *K*_A_ (Pearson correlation coefficient: 0.06 < *r* < 0.19; Fig. 3E). As shown in Fig. 3E, HM profile of duplicate genes in ORF diverges much quicker than that in promoters regions (Wilcoxon rank-sum test: *p*-value < 6.808E−10 for all gene modes), and ${D_{\rm HM - O}}\;$was more correlated with *K*_S_ and *K*_A_ (Pearson correlation: *r* = 0.18 and 0.19) than ${D_{\rm HM - P}}$ (Pearson correlation coefficient: *r* = 0.06 and 0.12). However, there is no significant difference in HM profile divergence between in ORF and promoter regions for randomized singleton genes (Wilcoxon rank-sum test: *p*-value = 0.8768). The result illustrated that the divergence of HM in ORF regions is more similar to the coding sequences divergence between paralog pairs.

We further explored the association of duplication modes with coding sequence divergence *D*_HM–O_ of WGDs tend to have a lower correlation with *K*_S_ and *K*_A_ than single-gene duplications (Fig. 3F; Fig. S4). Moreover, at similar *K*_S_ or *K*_A_ levels, transposed duplicate pairs were more likely to have higher HM divergence than the tandem and proximal duplicate gene pairs (Figs. 3A–3D). The different extent of HM divergence may be explained by the hypothesis that collinear duplicates tend to have similar chromatin environments, while transposed duplications re-locate to new chromosomal positions that usually have different chromatin environment (Wang et al., 2013).

### Relationship between epigenetic divergence and gene expression divergence between duplication gene pairs

**Figure 4:**
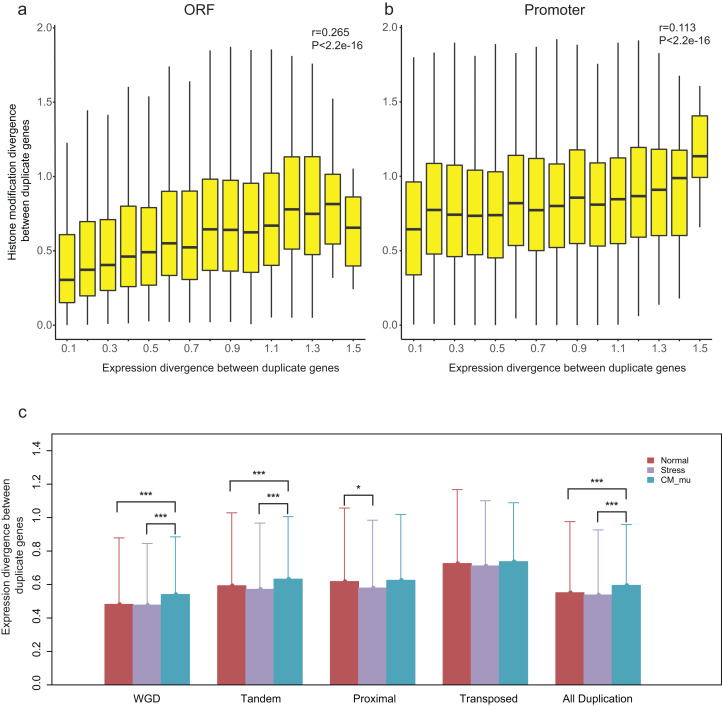
Relationship between HM divergence and expression divergence between paralog pairs, and the effect of different perturbation conditions on expression divergence of duplicate genes. (A and B) Boxplots show the relationship between HM divergence and expression divergence between duplicate genes in ORF and promoter regions. For other types of duplicate genes see Fig. S5. (C) The effects of different conditions on the expression divergence of paralog pairs with different types. Significant level of Wilcoxon rank sum test were shown as “***” for *P*-value < 0.001, and “*” for *P*-value < 0.05.

Our recent study revealed that epigenetic marks play a key role in determining transcriptional outcomes (Wang et al., 2015b). We thus asked whether the divergence of epigenetic patterns contributes to the expression divergence of paralogs. Correlation analysis was performed for HM divergence (${D_{\rm HM - P}}$ and ${D_{\rm HM - O}}$) and expression divergence (*E*) between paralog pairs, and we found that there is indeed a relation between them (Pearson correlation coefficient: *r* = 0.265 for ${D_{\rm HM - O}}$ and *r* = 0.113 for ${D_{\rm HM - P}}$, Figs. 4A and 4B). However, it is a weaker correlation between expression divergence and epigenetic divergence in promoter regions than that in ORF regions. Furthermore, the correlation differs among different modes of gene duplication (Pearson correlation coefficient: 0.239 < *r* < 0.368 for ${D_{\rm HM - O}}$ and 0.096 < *r* < 0.241 for ${D_{\rm HM - P}}$, Fig. S5). For proximal and transposed duplicates, there is a stronger correlation between HM divergence and expression divergence.

A previous study has suggested that histone modification enzymes and environmental stresses influence the expression evolution of duplicate genes in yeast (Zou et al., 2012). To test whether the expression divergence patterns of duplicate genes were consistent across different perturbation conditions in *A. thaliana*, we divided expression profiles into three types: Normal, Stress and CM_mu. The expression divergence between duplicate pairs under these three types of conditions were denoted as *E*_Normal_, *E*_Stress_ and ${E_{\rm CM\_mu}}$, respectively. Our results showed that the *E*_CM_nu_ is significantly greater than ${E_{\rm Normal}}$ and *E*_Stress_ (Fig. 4C, Wilcoxon rank-sum test: *p*-value < 2.2E−16). In contrast, there is no significant difference in expression divergence of duplicate genes in “Normal” and “Stress” conditions (Fig. 4C, Wilcoxon rank-sum test: *p*-value = 0.6097). As shown in Fig. 4C, different perturbation conditions have a similar effect on WGD and tandem duplicated genes with all duplicate genes but have little or no effect on the proximal and transposed duplicate genes. Taken together, epigenetic related enzymes (chromatin modifiers) may affect the expression evolution of duplicate genes, especially for WGD and tandem duplicated genes.

### Functional divergence and epigenetic divergence between duplicate pairs

**Figure 5:**
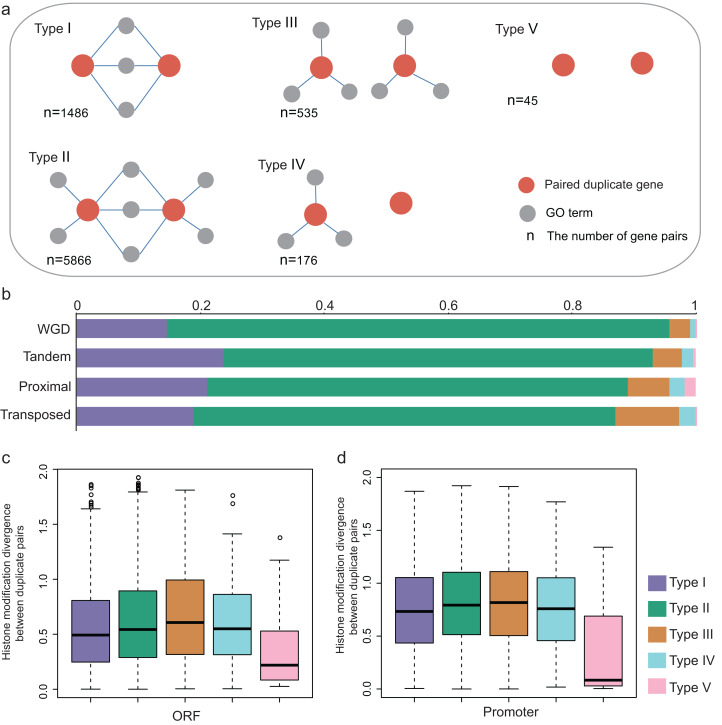
Functional divergence between paralog pairs. (A) Five types of functional divergence. (B) The relationship between functional divergence and duplication types. (C) The boxplots shows the relationship between functional divergence and HM divergence in ORF regions. (D) The boxplots showed the relationship between functional divergence and HM divergence in promoter regions.

The previous study suggested that duplicate pairs with different biological functions differ significantly in ORF HM and promoter HM profiles in yeast (Zou et al., 2012). Here, we estimated how different biological functions influence the epigenetic divergence between duplicate pairs in *A. thaliana*. The biological functional divergence is classified into five models based on the biological functions of the duplicate genes (Fig. 5A). For each duplicate gene pair (using A and B as an example), A and B share the same GO terms (Type I); A and B partially share GO terms (Type II); A and B do not share GO terms, but they both have associated GO terms (Type III), or just A has associated GO terms (Type IV), or both A and B do not have associated GO terms (Type V). Therefore, the extent of functional divergence showed the following trend: Type I ≈ Type V > Type II > Type III ≈ Type IV.

The relationship between functional divergence and duplication modes was investigated. We found that the WGD gene pairs were mostly enriched in Type II, and WGD gene pairs have higher functional divergence than single-gene pairs (Fisher test: *p*-value < 1.439E−14, Fig. 5B). The single-gene duplication shows the following functional divergence trend: Transposed > Proximal > Tandem (Fig. 5B).

We then investigated the relationship between functional divergence and HM divergence. Duplicate genes from Type I and Type V have lower HM divergence levels than Type II, III and IV (Figs. 5C and 5D; Wilcoxon rank-sum test: *p*-value < 0.005 for all cases). Type V duplicate genes have much lower HM divergence than Type I in both ORF and promoter regions (Wilcoxon rank-sum test: *p*-value < 0.005 for both cases). Besides, except for Type V, other types have higher HM divergence in promoter regions than in ORF regions. The results suggested duplicate genes with distinct biological processes undergo the different evolutionary rates of HM pattern in their ORF and promoter regions. That is, functional divergence is consistent with HM divergence.

**Figure 6:**
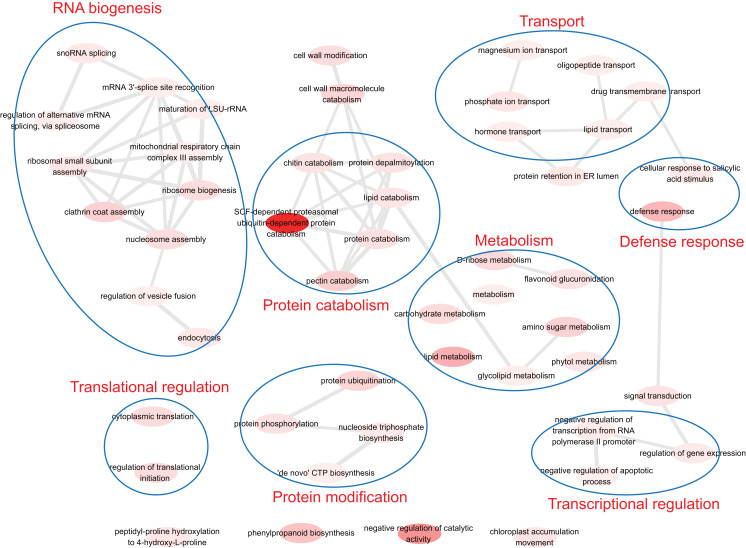
The functions of duplicate gene with high HM divergence. The enriched GO terms were involved in transcriptional/translational regulation, defense response, transport, protein modification, metabolism and so on.

To understand the biological function of duplicate genes, the functions of the duplicate gene with high HM divergence (${D_{\rm HM - O}}$ > 1 or *D*_HM–P_ > 1) were investigated. Using gene set enrichment analysis, eight functional models were identified: RNA biogenesis, translational regulation, protein catabolism, protein modification, transport, defense response, metabolism and transcriptional regulation (Fig. 6). It has been reported that the duplication of light-harvesting complex (LHC) in plants could diverge and acquire novel functions, such as response to various stress conditions including high light, high salinity, and nutrient limitation (Rochaix & Bassi, 2019). The biological functions associated with nutrient stress response, magnesium ion transport and phosphate ion transport were identified, indicating the validity of the results in this study.

## Discussion

This study overviewed the histone modification (HM) evolution of gene duplication, investigated the evolution’s effects on multiple biological points including HM pattern, sequence, expression and function in *A. thaliana*, and highlighted the important role of histone modification in an evolutionary context. To understand the HM of gene duplication comprehensively, we constructed the whole-genome HM profiling of ten common HM marks in five modes of gene duplication. Our study showed that WGD genes were mostly marked by active marks and H3K27me1/3, while transposed duplicate genes tended to be modified by H3K9me2. Besides, the correlations of HM marks between duplicate pairs illustrated positive relationships significantly, except for H3K9me2 marking WGD (Table 1). H3K9me2 is an HM mark which is strongly associated with transcriptional repression (Wang et al., 2008). Therefore, the active genes are likely to keep the same HM in the evolution of gene duplication. Furthermore, some marks, such as H3K4me2/3 and H3K36me3, showed co-occurrence between duplicate pairs (Figs. 1B–1F), which is consistent with our previous study (Wang et al., 2015b).

To explore whether the HM is different between duplicated gene pairs and non-duplicated genes, we have investigated the divergence of HM profiling. The result showed that duplicate gene pairs shared more similar HM patterns than randomized singleton pairs both in promoter regions and ORF regions. The same result has been found in yeast (Zou et al., 2012), which means the co-evolution between genetic and epigenetic is important in biology. Note that transposed duplicates have much higher HM divergence than other mode duplicates, which may illustrate the major role of transposon element in generating intergenic variation of species (Slotkin & Martienssen, 2007).

Previous studies have demonstrated that the evolution of epigenetic marks is coupled with coding sequence divergence after gene duplication in yeast and rice (Wang, Marowsky & Fan, 2014; Zou et al., 2012). Consistent with these observations, our data reveal further relationships between HM divergence and coding sequence divergence in *A. thaliana*. As expected, HM divergence is correlated with the sequence divergence between duplicate genes in the promoter and ORF regions (Figs. 2A–2D). At similar *K*_A_ or *K*_S_ levels, transposed duplicate genes tend to have a higher divergence of HM profile between duplicate genes than tandem and proximal duplicate genes. This may be explained by that collinear duplicates are more likely to have the same chromatin environments, while transposed duplicate genes re-locate to new chromosomal locations that often have different chromatin environments (Wang et al., 2013).

The expression analysis revealed that HM divergence and expression divergence between duplicate genes are significantly correlated, which illustrates epigenetic marks have an important role in the regulation of gene expression in eukaryotic genomes. Notably, for proximal and transposed duplicates, there is a strong correlation between HM divergence and expression divergence. As a proof of concept, we validated our result using RNA-seq datasets through collecting three types of expression profiles: Normal, Stress, and Mutated (CM_mu). Our results showed that expression divergence between duplicate pairs in “CM_mu” condition is significantly greater than the other two conditions (Wilcoxon rank-sum test: *p*-value < 2.2E−16). Epigenetic related enzymes may play an important role in the expression evolution of duplicate genes.

Lastly, we investigated the functional divergence of duplicated genes with the epigenetic divergence. Results show that functionally selective constraints may impose on epigenetic evolution after gene duplication. Furthermore, gene duplicate modes showed the following functional divergence: WGD > Transposed > Proximal > Tandem. According to the result, we suppose that functionally selective constraints pose a greater influence on the epigenetic pattern divergence of WGD genes. It has reported that the WGD could cause a diversification of gene repertoires and allowed functional specialization of its copies in vertebrates, which increased the complexity of gene regulation (Marletaz et al., 2018).

The GO-based analysis also found that duplicate genes with high HM divergence are involved in transcriptional/translational regulation and stress defense response, which is consistent with previous studies (Hanada et al., 2008; Maere et al., 2005). In the future, we hope that studying the three-dimensional (3D) regulation of duplicate genes will enhance our understanding of the mechanisms of gene duplication.

## Conclusions

It has been reported that genome duplication contributes to the histone modification status change (Zhang et al., 2019), then the histone modification could regulate the gene expression backforward. This study investigated the characterizations of duplicate genes and described the features of duplicate genes with different histone modifications. We found that duplicate genes share more similar HM patterns than randomized singleton pairs in their promoters and ORF regions. Although it is not clear to what extent the epigenetic profiles are the cause or effect of duplicate genes, our result suggests that genetic variables could give rise to epigenetic divergence. Besides, this study shows that the correlation between HM divergence and the expression divergence of duplicate pairs is significant. The expression divergence between duplicate genes changes with the mutation of chromatin modifiers. Based on the results, the functional divergence has a role in the epigenetic divergence after gene duplication, and duplicate genes with high HM divergence are involved in multiple biological processes. In summary, this work will shed light on the role of epigenetic factors contributing to the regulatory evolution and divergence of gene duplication.

## Supplemental Information

10.7717/peerj.10426/supp-1 Supplemental Information 1 RNA-seq samples and ChIP-chip datasets.

10.7717/peerj.10426/supp-2 Supplemental Information 2 Duplicate gene pairs identified in this study.

10.7717/peerj.10426/supp-3 Supplemental Information 3 Enrichment of histone modification marks.

10.7717/peerj.10426/supp-4 Supplemental Information 4 The description of 10 histone modification marks.

10.7717/peerj.10426/supp-5 Supplemental Information 5 The GO terms of the Arabidopsis genes used in the analysis.

## Additional Information and Declarations

### Data Availability

The following information was supplied regarding data availability: All supporting RNA-seq data, 88 RNA-seq samples from 16 publications, duplicate gene pairs and the histone mark enrichment are available in the Supplemental Files. All histone modification data are available in the Gene Expression Omnibus (GEO): GSE13613, GSE24710, GSE83606 and GSE54489.

## References

[ref-1] Berke L, Sanchez-Perez GF, Snel B (2012). Contribution of the epigenetic mark H3K27me3 to functional divergence after whole genome duplication in Arabidopsis. Genome Biology.

[ref-2] Berke L, Snel B (2014). The histone modification H3K27me3 is retained after gene duplication and correlates with conserved noncoding sequences in Arabidopsis. Genome Biology and Evolution.

[ref-3] Chen ZJ (2007). Genetic and epigenetic mechanisms for gene expression and phenotypic variation in plant polyploids. Annual Review of Plant Biology.

[ref-4] Cusack BP, Wolfe KH (2007). Not born equal: increased rate asymmetry in relocated and retrotransposed rodent gene duplicates. Molecular Biology and Evolution.

[ref-5] Flagel LE, Wendel JF (2009). Gene duplication and evolutionary novelty in plants. New Phytologist.

[ref-6] Freeling M (2009). Bias in plant gene content following different sorts of duplication: tandem, whole-genome, segmental, or by transposition. Annual Review of Plant Biology.

[ref-7] Hanada K, Zou C, Lehti-Shiu MD, Shinozaki K, Shiu SH (2008). Importance of lineage-specific expansion of plant tandem duplicates in the adaptive response to environmental stimuli. Plant Physiology.

[ref-8] Hu Z, Han Z, Song N, Chai L, Yao Y, Peng H, Ni Z, Sun Q (2013). Epigenetic modification contributes to the expression divergence of three *TaEXPA1* homoeologs in hexaploid wheat (Triticum aestivum). New Phytologist.

[ref-9] Huang DW, Sherman BT, Lempicki RA (2009). Systematic and integrative analysis of large gene lists using DAVID bioinformatics resources. Nature Protocols.

[ref-10] Keller TE, Yi SV (2014). DNA methylation and evolution of duplicate genes. Proceedings of the National Academy of Sciences of the United States of America.

[ref-11] Kim D, Pertea G, Trapnell C, Pimentel H, Kelley R, Salzberg SL (2013). TopHat2: accurate alignment of transcriptomes in the presence of insertions, deletions and gene fusions. Genome Biology.

[ref-12] Kustatscher G, Grabowski P, Schrader TA, Passmore JB, Schrader M, Rappsilber J (2019). Co-regulation map of the human proteome enables identification of protein functions. Nature Biotechnology.

[ref-13] Liao X, Bao H, Meng Y, Plastow G, Moore S, Stothard P (2014). Sequence, structural and expression divergence of duplicate genes in the bovine genome. PLOS ONE.

[ref-14] Maere S, De Bodt S, Raes J, Casneuf T, Van Montagu M, Kuiper M, Van de Peer Y (2005). Modeling gene and genome duplications in eukaryotes. Proceedings of the National Academy of Sciences of the United States of America.

[ref-15] Mantel N (1967). The detection of disease clustering and a generalized regression approach. Cancer Research.

[ref-16] Marletaz F, Firbas PN, Maeso I, Tena JJ, Bogdanovic O, Perry M, Wyatt CDR, De la Calle-Mustienes E, Bertrand S, Burguera D, Acemel RD, van Heeringen SJ, Naranjo S, Herrera-Ubeda C, Skvortsova K, Jimenez-Gancedo S, Aldea D, Marquez Y, Buono L, Kozmikova I, Permanyer J, Louis A, Albuixech-Crespo B, Le Petillon Y, Leon A, Subirana L, Balwierz PJ, Duckett PE, Farahani E, Aury JM, Mangenot S, Wincker P, Albalat R, Benito-Gutierrez E, Canestro C, Castro F, D’Aniello S, Ferrier DEK, Huang S, Laudet V, Marais GAB, Pontarotti P, Schubert M, Seitz H, Somorjai I, Takahashi T, Mirabeau O, Xu A, Yu JK, Carninci P, Martinez-Morales JR, Crollius HR, Kozmik Z, Weirauch MT, Garcia-Fernandez J, Lister R, Lenhard B, Holland PWH, Escriva H, Gomez-Skarmeta JL, Irimia M (2018). Amphioxus functional genomics and the origins of vertebrate gene regulation. Nature.

[ref-17] Otto SP, Whitton J (2000). Polyploid incidence and evolution. Annual Review of Genetics.

[ref-18] Rochaix JD, Bassi R (2019). LHC-like proteins involved in stress responses and biogenesis/repair of the photosynthetic apparatus. Biochemical Journal.

[ref-19] Slotkin RK, Martienssen R (2007). Transposable elements and the epigenetic regulation of the genome. Nature Reviews Genetics.

[ref-20] Sui Y, Li B, Shi J, Chen M (2014). Genomic, regulatory and epigenetic mechanisms underlying duplicated gene evolution in the natural allotetraploid Oryza minuta. BMC Genomics.

[ref-21] Supek F, Bosnjak M, Skunca N, Smuc T (2011). REVIGO summarizes and visualizes long lists of gene ontology terms. PLOS ONE.

[ref-22] Trapnell C, Williams BA, Pertea G, Mortazavi A, Kwan G, van Baren MJ, Salzberg SL, Wold BJ, Pachter L (2010). Transcript assembly and quantification by RNA-seq reveals unannotated transcripts and isoform switching during cell differentiation. Nature Biotechnology.

[ref-34] Trapnell C, Roberts A, Goff L, Pertea G, Kim D, Kelley DR, Pimentel H, Salzberg SL, Rinn JL, Pachter L (2012). Differential gene and transcript expression analysis of RNA-seq experiments with TopHat and Cufflinks. Nature Protocols.

[ref-23] Wang S, Adams KL (2015). Duplicate gene divergence by changes in microRNA binding sites in Arabidopsis and Brassica. Genome Biology and Evolution.

[ref-24] Wang H, Beyene G, Zhai J, Feng S, Fahlgren N, Taylor NJ, Bart R, Carrington JC, Jacobsen SE, Ausin I (2015a). CG gene body DNA methylation changes and evolution of duplicated genes in cassava. Proceedings of the National Academy of Sciences of the United States of America.

[ref-25] Wang J, Marowsky NC, Fan C (2014). Divergence of gene body DNA methylation and evolution of plant duplicate genes. PLOS ONE.

[ref-26] Wang J, Meng X, Yuan C, Harrison AP, Chen M (2015b). The roles of cross-talk epigenetic patterns in *Arabidopsis thaliana*. Briefings in Functional Genomics.

[ref-27] Wang Y, Tan X, Paterson AH (2013). Different patterns of gene structure divergence following gene duplication in Arabidopsis. BMC Genomics.

[ref-28] Wang Y, Wang X, Lee TH, Mansoor S, Paterson AH (2013). Gene body methylation shows distinct patterns associated with different gene origins and duplication modes and has a heterogeneous relationship with gene expression in *Oryza sativa* (rice). New Phytologist.

[ref-29] Wang Y, Wang X, Paterson AH (2012). Genome and gene duplications and gene expression divergence: a view from plants. Annals of the New York Academy of Sciences.

[ref-30] Wang Z, Zang C, Rosenfeld JA, Schones DE, Barski A, Cuddapah S, Cui K, Roh TY, Peng W, Zhang MQ, Zhao K (2008). Combinatorial patterns of histone acetylations and methylations in the human genome. Nature Genetics.

[ref-31] Zhang H, Zheng R, Wang Y, Zhang Y, Hong P, Fang Y, Li G, Fang Y (2019). The effects of Arabidopsis genome duplication on the chromatin organization and transcriptional regulation. Nucleic Acids Research.

[ref-32] Zhao M, Meyers BC, Cai C, Xu W, Ma J (2015). Evolutionary patterns and coevolutionary consequences of MIRNA genes and microRNA targets triggered by multiple mechanisms of genomic duplications in soybean. Plant Cell.

[ref-33] Zou Y, Su Z, Huang W, Gu X (2012). Histone modification pattern evolution after yeast gene duplication. BMC Evolutionary Biology.

