# Supplementary material for: In situ dissecting the evolution of gene duplication with different histone modification patterns based on high-throughput data analysis in Arabidopsis thaliana
Source: PeerJ. 2021 Jan 5;9:e10426. doi: 10.7717/peerj.10426 (PMC7792519; doi:10.7717/peerj.10426)
Supplement: Supplemental Information 3 [file peerj-09-10426-s003.pdf]

Additional file 1

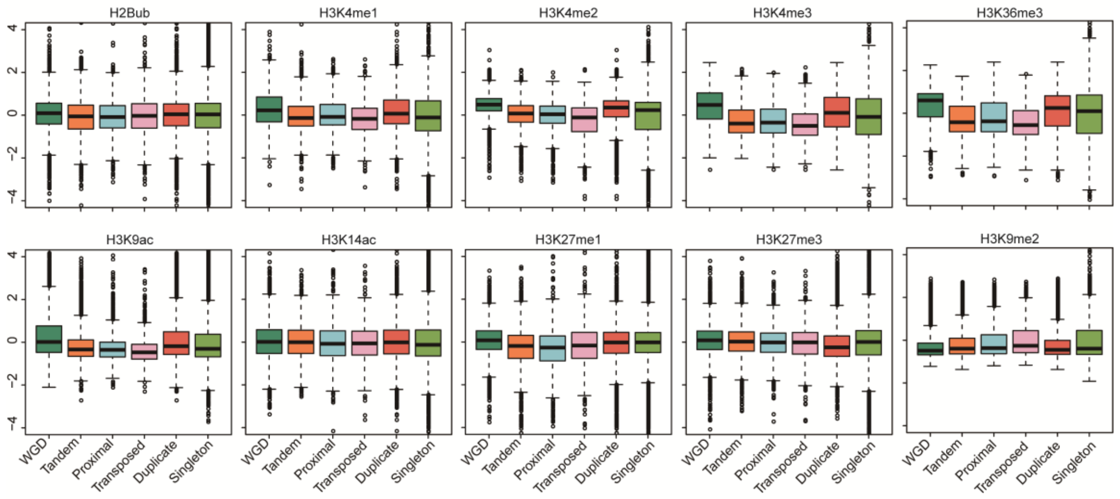

Fig. S1 Comparison the enriched levels of ten histone modification marks among four types duplicate genes, singleton and all duplicate *Arabidopsis* genes in ORF regions.

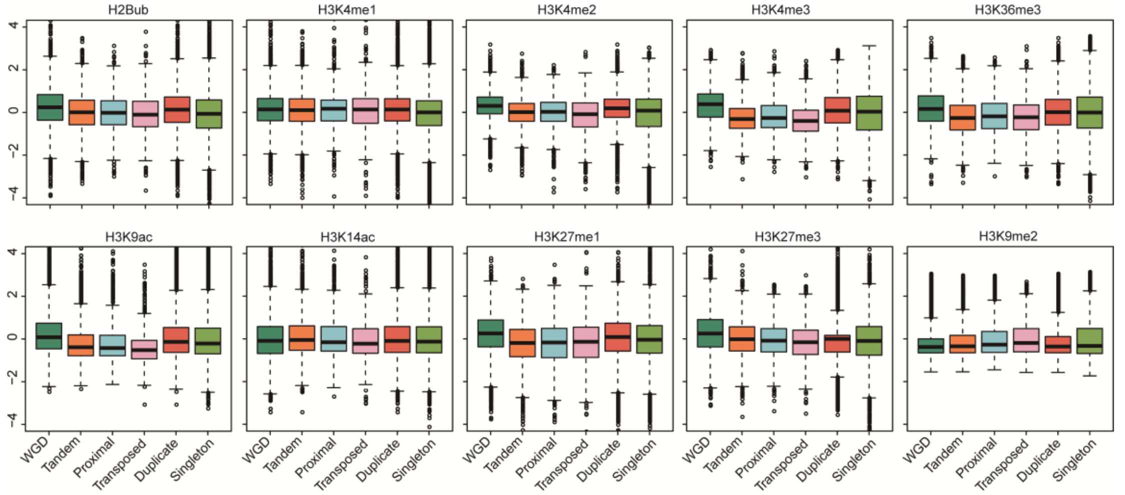

Fig. S2 Comparison the enriched levels of ten histone modification marks among four types duplicate genes, singleton and all duplicate *A. thaliana* genes in promoter regions.

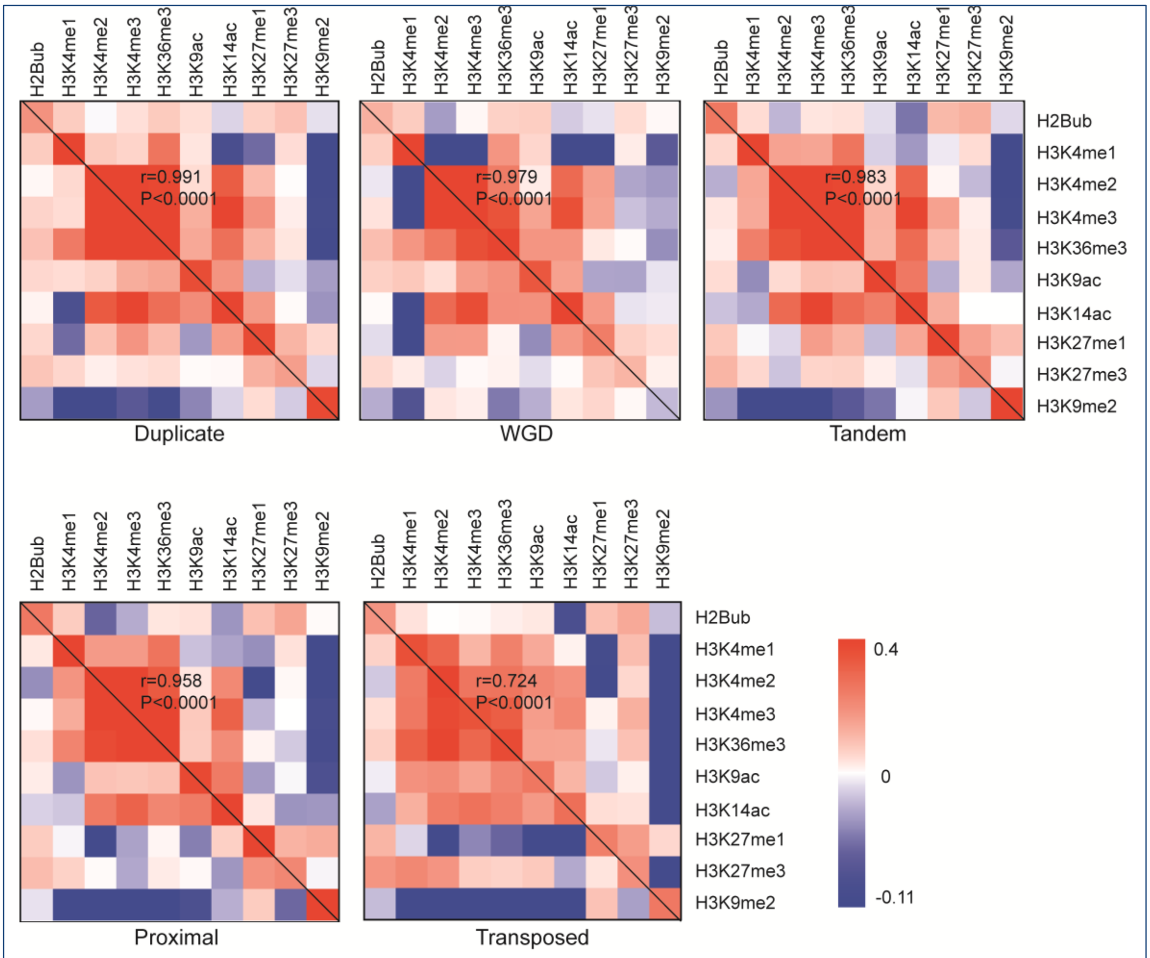

Fig. S3 Heatmap matrix of pairwise Pearson correlation of histone modification marks for different types of paralog pairs in promoter regions.

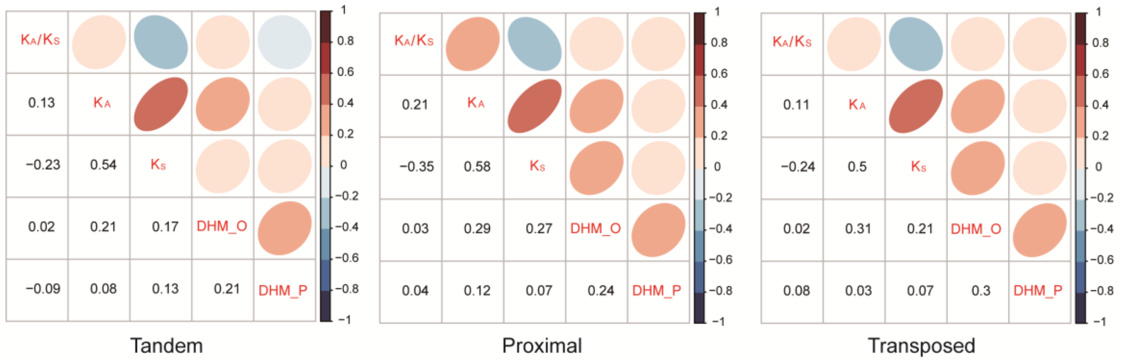

Fig. S4 Correlation matrix between histone modification divergence and sequence divergence for tandem, proximal and transposed duplicate genes.

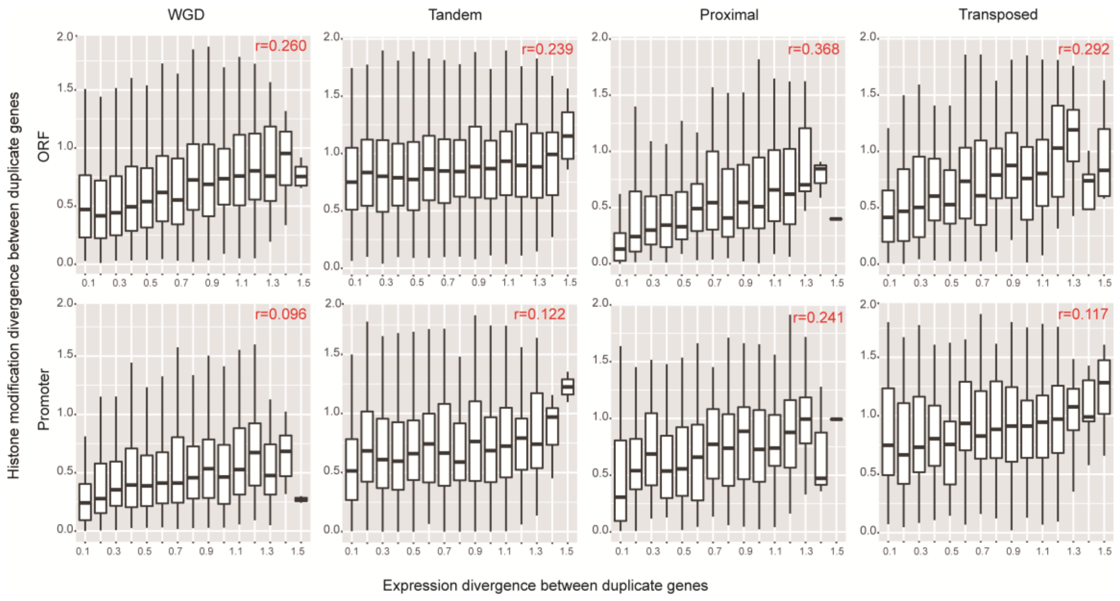

Fig. S5 Relationship between ORF/promoter histone modification divergence and expression divergence between different types of paralog pairs.
