# Supplementary material for: In situ dissecting the evolution of gene duplication with different histone modification patterns based on high-throughput data analysis in Arabidopsis thaliana
Source: PeerJ. 2021 Jan 5;9:e10426. doi: 10.7717/peerj.10426 (PMC7792519; doi:10.7717/peerj.10426)
Supplement: Supplemental Information 4 [file peerj-09-10426-s004.docx]

**The description of 10 histone modification mark in “In situ dissecting the evolution of gene duplication with different histone modification patterns based on high-throughput data analysis in *Arabidopsis thaliana*”**

The epigenetic regulation include histone modification (HM), DNA methylation and histone variants. Histone modification is a covalent post-translational modification (PTM) to histone proteins and the PTMs made to histones can impact gene expression by altering chromatin structure or recruiting histone modifiers. In most species, histone proteins contain four types: H2A, H2B, H3, and H4, these histones could be modified by acetylation, methylation, phosphorylation, and ubiquitylation.

For example, histone H3 is primarily acetylated (ac) at lysines (K) 9, 14, 18, 23, and 56, methylated (me) at lysines (K) 4, 9, 27, 36, and 79, and phosphorylated at ser10, ser28, Thr3, and Thr11. H3K4me1 is an epigenetic modification to protein Histone H3 and it is a mark that indicates the mono-methylation at the 4th lysine residue of H3. The histone modification is an important kind of epigenetic marks and could affect the gene transcriptional activation. Some marks locate nearby active genes, such as H3K4me1/2/3, H3K9ac, H3K14ac and H3K36me3, are called active marks, and some marks locate nearby repressive gene, such as H3K27me1/3, are called repressive marks.

H2Bub: a monoubiquitination at a specific lysine on histone H2B.

H3K4me1: an epigenetic modification to the protein Histone H3. It is a mark that indicates the mono-methylation at the 4th lysine residue of the histone H3 protein and often associated with gene enhancers.

H3K4me2: an epigenetic modification to the protein Histone H3. It is a mark that indicates the di-methylation at the 4th lysine residue of the histone H3 protein.

H3K4me3: an epigenetic modification to the protein Histone H3. It is a mark that indicates the tri-methylation at the 4th lysine residue of the histone H3 protein and often involved in the regulation of gene expression.

H3K9ac: an epigenetic modification to the protein Histone H3. It is a mark that indicates the acetylation at the 9th lysine residue of the histone H3 protein.

H3K9me2: an epigenetic modification to the protein Histone H3. It is a mark that indicates the di-methylation at the 9th lysine residue of the histone H3 protein.

H3K27me1: an epigenetic modification to protein Histone H3. It is a mark that indicates the mono-methylation at the 27th lysine residue of the histone H3 protein.

H3K27me3: an epigenetic modification to the protein Histone H3. It is a mark that indicates the tri-methylation at the 27th lysine residue of the histone H3 protein.

H3K36me3: an epigenetic modification to the protein Histone H3. It is a mark that indicates the tri-methylation at the 36th lysine residue of the histone H3 protein.

H3K14ac：an epigenetic modification to the protein Histone H3. It is a mark that indicates the acetylation at the 14th lysine residue of the histone H3 protein
